# Role of *GSTM1* and *GSTT1* genotypes in differentiated thyroid cancer and interaction with lifestyle factors: Results from case-control studies in France and New Caledonia

**DOI:** 10.1371/journal.pone.0228187

**Published:** 2020-01-30

**Authors:** Catherine Tcheandjieu, Emilie Cordina-Duverger, Claire Mulot, Dominique Baron-Dubourdieu, Anne-Valérie Guizard, Claire Schvartz, Pierre Laurent-Puig, Pascal Guénel, Thérèse Truong

**Affiliations:** 1 Université Paris-Saclay, UVSQ, INSERM, CESP, Villejuif, France; 2 Centre de Recherche des Cordeliers, INSERM, Sorbonne Université, USPC, Université Paris Descartes, Université Paris Diderot, EPIGENETEC, Paris, France; 3 Laboratory of Anatomy and Cytopathology, Noumea, New Caledonia, France; 4 Registre Général des Tumeurs du Calvados, Centre François Baclesse, Caen, France; 5 U1086 INSERM–UCN “ANTICIPE”, Caen, France; 6 Registre spécialisé des Cancers de la Thyroide Marne-Ardennes Institut GODINOT, Reims, France; INSERM, FRANCE

## Abstract

**Background:**

*GSTM1* and *GSTT1* are involved in detoxification of xenobiotics, products of oxidative stress and in steroid hormones metabolism. We investigated whether *GSTM1* and *GSTT1* gene deletion was associated with DTC risk and explored interaction with non-genetic risk factors of DTC.

**Methods:**

The study included 661 DTC cases and 736 controls from two case-control studies conducted in France and New Caledonia. Odds ratios (OR) and their confidence interval (CI) for DTC associated with GST genotypes, alcohol drinking, tobacco smoking, body mass index and hormonal factors were calculated using logistic regression models.

**Results:**

Results are presented for Europeans and Melanesians combined, as no heterogeneity between groups was detected. We found that DTC risk increased with obesity and decrease with alcohol drinking. After stratification by gene deletion status, the OR for obesity was 5.75, (95%CI 2.25–14.7) among individuals with *GSTT1* and *GSTM1*-deleted genotype, and 1.26, (95%CI 0.89–1.77) in carriers of both genes (p-interaction = 0.02). The OR for drinking ≥1 glass/week was 0.33 (95%CI 0.15–0.74) in *GSTT1*-null individuals while it was 1.01 (95%CI 0.67–1.52) in non-null carriers of the gene (p-interaction = 0.01). No interaction between GST genotypes and other non-genetic risk factors was detected.

**Conclusion:**

*GSTM1* and *GSTT1* genotypes may modulate the DTC risk associated with BMI and alcohol consumption.

## Introduction

Differentiated thyroid carcinoma (DTC) accounts for about 90% of all thyroid cancers. The incidence of thyroid cancer is characterized by 4–5 times higher rates in women than in men and considerable ethnic and geographic variation [1,2]. Particularly high incidence rates were observed in Melanesian women of New Caledonia, South Pacific (71.4/100 000 person-years (py) in 1995–1999 [1–3], contrasting with rates ranging from 2 to 8/100 000 py among women in other high income countries [2]). During the last decades, the incidence of DTC has increased regularly in most high-resource countries. Part of this increase has been attributed mainly to changes in medical screening practices that enhance the detection of small dormant microcarcinomas [4–6], but changes in environmental or lifestyle factors may also contribute to this increase [5].

Apart from exposure to ionizing radiation during childhood, a well-established risk factor for DTC, increased DTC risk has also been associated with overweight, iodine deficiency, high parity, and late age at menarche. By contrast, several studies have reported that the risk of DTC decreased with alcohol or tobacco consumption [7–11]. Thyroid cancer is one of the cancers with the highest familial risk [12], suggesting a role of genetic risk factors. However, only a few variants have been identified thus far [13] and, to the best of our knowledge, no gene-environment interactions have been convincingly reported for DTC.

Glutathione S-transferase (GST) genes, such as *GSTM1* and *GSTT1*, encode for phase II enzymes, and are involved in the steroid hormone metabolism and in the detoxification of various xenobiotics and products of oxidative stress. The enzyme activity depends on the number of copies of *GSTM1* and *GSTT1* (copy number variation, CNV) in the genome [14,15], with complete gene deletion resulting in a loss of function. It can thus be hypothesized that gene deletion confers higher vulnerability to carcinogen exposure [16].

In a meta-analysis of 12 studies conducted in countries from Europe, Asia and South America that investigated the association between DTC and *GSTM1* or *GSTT1* genotypes, significant heterogeneity between studies was reported [17]. Heterogeneity may be explained by unmeasured exposures in endogenous or exogenous risk factors that modify the association of DTC with GST genotypes.

In the present paper, we used data collected in studies on DTC conducted in populations of European origin in France and in populations of European and Melanesian origins form New Caledonia, to investigate the role of G*STM1* and *GSTT1* genotypes in DTC risk, and their interaction with suspected risk factors of DTC that could be modulated by these genes such as hormonal factors, cigarette smoking and alcohol drinking, and obesity.

## Material and methods

### Study population

We used data from two case-control studies on thyroid cancer conducted in metropolitan France (CATHY study) and in New Caledonia (NC study). All participants provided signed informed consent. The study was approved by the review board of the French institute of health and medical research (INSERM) and authorized by the French data protection authority (CNIL).

The CATHY study [18] is a population-based case-control study performed in three French “*départements*” (Marne, Ardennes, and Calvados) covered by a cancer registry. Cases were patients living in these areas aged 25 years and older diagnosed with DTC between 2002 and 2007. Controls were selected at random using the telephone directory and unlisted phone numbers of all private homes in the study areas, and were frequency-matched to the cases by 5-year age group and study area. To prevent possible selection bias arising from differential participation rates across categories of socioeconomic status (SES), the control group was selected to reflect the distribution by SES categories of the general population, as described in details previously [18]. From 621 cases and 706 controls recruited for the study, saliva DNA samples (Oragen®) were obtained for 482 cases and 565 controls of self-declared European ancestry.

The NC study is a country-wide, population-based, case-control study [3,7,19]. The cases included patients with DTC diagnosed between 1993 and 1999 who had been living in NC for at least 5 years at the time of diagnosis. The cases were identified from the two pathology laboratories in NC and from active searches in the medical records of the main hospitals. Age- and sex-matched controls were randomly selected from recently updated electoral rolls. A total of 332 cases and 412 controls were included; 42 cases and 133 controls self-declared Europeans and 206 cases and 156 controls self-declared Melanesians. Saliva DNA samples (Oragen®) were available for 284 Melanesians (164 cases and 120 controls) and 108 Europeans (27 cases and 81 controls).

In the two studies, information on ethnicity, personal and familial history of thyroid disease, gynecological and reproductive history, anthropometric factors, diet, alcohol intake, tobacco smoking, and residential and occupational histories was collected during in-person interviews by trained interviewers. BMI was defined as weight (in kilograms) divided by height (in meters) squared. Alcohol drinking was assessed as the lifetime average number of glasses per week using information from the questionnaire for each type of beverage separately (beer, wine, aperitif, and liqueur). Because the ethanol content is approximately the same for an ordinary glass of any alcoholic beverage, the total number of drinks per week was used as an indicator of total alcohol intake. Pack-years of cigarette smoking were calculated from the total number of years of tobacco smoking and from the number of cigarettes smoked per day for each smoking period.

Women were considered to be users of oral contraceptives if they had ever taken pills for at least 6 months. Women were considered postmenopausal if they reported no menstruation for at least 1 year or used menopausal hormone therapy (MHT) (natural menopause), or if they had bilateral ovariectomy (artificial menopause). Women with unknown menopausal status, because of hysterectomy before cessation of menstruations or unknown date of last menstruation, were considered postmenopausal if they were 50 years old or more (the median age at menopause in women with natural menopause).

Association between DTC risk and reproductive factors, BMI, tobacco smoking and alcohol drinking were analyzed in detail previously in the NC [7, 19] and CATHY studies [18]. In the present paper, the analyses were performed in the subset of individuals with genotype data from these two studies.

### Genotyping

DNA extracted from saliva samples was used to determine the copy number variation (CNV) of *GSTM1* and *GSTT1* using TaqMan gene copy number detection designed by Applied Biosystems. The genotyping was processed by Integragen (Evry, France). Real-time PCR was run on an Applied Biosystems 7900HT Fast system with gene-specific primers for *GSTM1* probes (Hs02575461_cn) and *GSTT1* probes (Hs00010004_cn), along with primer for the RNase P gene as a reference. Each sample was run in triplicate using 50 ng of genomic DNA. The gene-specific primers were validated in 90 CEPH individuals and genotyping carried out blindly as to case-control status. CopyCaller software V1 (Applied Biosystems) was used to quantify the number of copies in each sample.

Thirty-one subjects (5 cases and 26 controls) in the CATHY study and 14 subjects (8 cases and 6 controls) in the NC study had missing genotypes for both *GSTM1* and *GSTT1* and were excluded from the analysis. Therefore, a total 1124 Europeans (504 cases and 620 controls) and 270 Melanesians (156 cases and 114 controls) were included in the analyses.

### Statistical analysis

Odds ratios (ORs) were calculated for *GSTM1* and *GSTT1* genotypes using unconditional logistic regression, comparing categories defined by the gene copy number coded as a categorical variable (0, 1, or ≥2 copies) and adjusting for age (5-year intervals), sex, and area of residence. We also compared non-carriers (null genotype) to carriers of at least one copy of the gene (non-null genotype). We first analyzed Europeans and Melanesians separately and tested the heterogeneity of the ORs using the Cochran’s Q test or likelihood ratio test. As no heterogeneity was detected, the combined dataset was used in further analyses (additionally adjusted for ethnic group).

We tested the interaction between *GSTM1* and *GSTT1* genotypes and tobacco smoking, alcohol intake, BMI, and hormonal and reproductive factors using the likelihood ratio test, comparing models with and without the interaction term. Tobacco smoking, alcohol intake, and BMI were analyzed in men and women together, whereas hormonal and reproductive factors were analyzed in women only. To account for the potential confounding effect of hormonal and reproductive factors, we also performed the analysis for tobacco smoking, alcohol drinking, and BMI in women separately. The results were similar to those obtained for men and women combined and are not shown.

## Results

The characteristics of the study sample are presented in Table 1 for Europeans and Melanesians separately. Cases and controls had similar sex and age distributions in each group. Among cases, the proportion of papillary thyroid cancers was similar in the two groups (~85%).

**Table 1:** Selected characteristics of the study sample by ethnic group.

	Europeans		Melanesians	
	Cases N = 504 n (%)	Controls N = 620 n (%)	Cases N = 156 n (%)	Controls N = 114 n (%)
**Mean age (years)**	51.3	51.0	45.6	46.0
**Sex**	** **	** **		
Women	402 (79.76)	463 (74.68)	148 (94.87)	105 (92.11)
Men	102 (20.24)	157 (25.32)	8 (5.13)	9 (7.89)
**Study population**				
CATHY	477 (94.64)	539 (86.94)		
New Caledonia	27 (5.36)	81 (13.06)	156 (100)	114 (100)
**Area of residence in CATHY study**				
Calvados	151 (29.96)	192 (30.97)		
Marne	217 (43.06)	250 (40.32)		
Ardennes	109 (21.63)	97 (15.65)		
**Area of residence in NC study**				
North	3 (11.11)	6 (7.41)	35 (22.44)	29 (25.44)
South	23 (85.19)	74 (91.36)	54 (34.62)	46 (40.35)
Loyalty Islands	1 (3.70)	1 (1.23)	67 (42.95)	39 (34.21)
**Histological type of cancer**				
Papillary	444 (88.10)		132 (84.62)	
Follicular	60 (11.90)		24 (15.38)	

Obesity (BMI ≥ 30 kg/m2) and high parity were more prevalent in Melanesians than in Europeans. Conversely, alcohol drinking and use of oral contraceptives were less common in Melanesians (Table 2). However odds ratios calculated in Europeans and Melanesians were comparable and none of the p-values for heterogeneity was significant. In the analysis of the two groups combined, thyroid cancer risk was increased in obese subjects and decreased with alcohol consumption. No association with tobacco smoking was observed. Among women, late age at menarche was associated with a non-significant increased risk of DTC.

**Table 2:** Association between a selection of lifestyle, hormonal and reproductive factors and DTC risk by ethnic group.

	Europeans			Melanesians			Europeans and Melanesians			Het^d^
	Cases (%)	Controls(%)	OR 95% CI	Cases (%)	Controls(%)	OR 95% CI	Cases n (%)	Controls (%)	OR 95% CI	p-value
**Men and women**	**N = 498**^**c**^	**N = 603**^**c**^	*** ***	**N = 151**^**c**^	**N = 105**^**c**^	** **	**N = 649**	**N = 708**	** **	** **
**BMI**^**a**^**, kg/m^2^**			* *							0.21
<25	236 (47.39)	319 (52.90)	Ref	37 (24.50)	37 (35.24)	Ref	273 (42.06)	356 (50.28)	Ref	
25–29.9	159 (31.93)	283 (30.35)	1.26 0.93–1.69	52 (34.44)	40 (38.10)	1.01 0.48–2.12	211 (32.51)	223 (31.50)	1.27 0.97–1.66	
≥30	103 (20.68)	101 (16.75)	1.32 0.93–1.88	62 (41.06)	28 (26.67)	2.77 1.30–5.93	165 (25.42)	129 (18.22)	1.62 1.19–2.20	
**Alcohol drinking**^**a**^**, glass/week**										
Never	140 (28.11)	127 (21.06)	Ref	68 (45.03)	42 (40.00)	Ref	208 (32.05)	169 (23.87)	Ref	
<1	248 (49.80)	326 (54.06)	0.67 0.49–0.92	64 (42.38)	51 (48.57)	0.53 0.27–1.01	312 (48.07)	377 (53.25)	0.65 0.50–0.86	
≥1	110 (22.09)	150 (24.88)	0.70 0.47–1.04	19 (12.58)	12 (11.43)	0.78 0.29–2.13	129 (19.88)	162 (22.88)	0.70 0.49–0.99	
**Cigarette smoking**^**a**^**, pack-years**										
Never	268 (53.82)	317 (52.57)	Ref	91 (60.26)	64 (60.95)	Ref	359 (55.32)	381 (53.81)	Ref	
<20	164 (32.93)	188 (31.18)	1.12 0.84–1.50	41 (27.15)	24 (22.86)	1.21 0.58–2.51	205 (31.59)	212 (29.94)	1.18 0.90–1.53	
≥20	66 (13.25)	98 (16.25)	0.80 0.53–1.21	19 (12.58)	17 (16.19)	1.19 0.50–2.82	85 (13.10)	115 (16.24)	0.85 0.60–1.22	
**Women**	**N = 369**	**N = 382**	** **	**N = 126**	**N = 87**	** **	**N = 495**	**N = 469**	** **	** **
**Age at menarche**^**b**^										0.61
≤13 years	306 (82.93)	328 (85.86)	Ref	59 (46.83)	49 (56.32)	Ref	365 (73.74)	377 (80.38)	Ref	
>13 years	63 (17.07)	54 (14.14)	1.43 0.93–2.2	67 (53.17)	38 (43.68)	1.23 0.64–2.37	130 (26.26)	92 (19.62)	1.35 0.95–1.91	
**Oral contraception use**^**b**^										
Never	101 (27.37)	87 (22.77)	Ref	92 (73.02)	64 (73.56)	Ref	193 (38.99)	151 (32.20)	Ref	
Ever	268 (72.63)	295 (77.23)	0.76 0.49–1.18	34 (26.98)	23 (26.44)	0.93 0.42–2.07	302 (60.01)	318 (67.80)	0.84 0.58–1.22	
**Number of full-term pregnancies**^**b**^										
0	30 (8.13)	42 (10.99)	Ref	16 (12.03)	10 (11.11)	Ref	46 (9.16)	52 (11.02)	Ref	
1–2	207 (56.10)	210 (54.97)	1.49 0.84–2.67	28 (21.05)	26 (28.89)	0.95 0.28–3.20	235 (46.81)	236 (50.00)	1.43 0.86–2.38	
≥3	132 (35.77)	130 (34.03)	1.45 0.78–2.69	82 (61.65)	51 (56.67)	0.98 0.30–3.15	214 (42.63)	181 (38.35)	1.46 0.86–2.49	
**Menopausal status**^**b**^										
Pre-menopause	156 (42.28)	201 (52.62)	Ref	84 (66.67)	57 (65.52)	Ref	240 (48.48)	258 (55.01)	Ref	
Post-menopause	213 (57.72)	181 (47.38)	1.7 0.94–3.09	42 (33.33)	30 (34.48)	1.09 0.35–3.37	255 (51.52)	211 (44.99)	1.33 0.83–2.13	

^a^ The multivariate regression model includes age, sex, area of residence, ethnic group, BMI, alcohol consumption, and cigarette smoking.

^b^ The multivariate regression model includes age, area of residence, ethnic group, BMI, alcohol consumption, cigarette smoking, age at menarche, number of full-term pregnancies, and oral contraception.

^c^ The sum does not add up to the total (504 cases/620 controls in European, 156 cases/114 controls in Melanesian) because of missing values for some variables in the multivariate analysis.

^d^ Test for heterogeneity of odds ratios across ethnic groups

OR: odds ratio, CI: confidence interval

The proportion of control subjects with a ‘null’ genotype was much higher in Melanesians than in Europeans (82.46% vs 48.06% for GSTM1; 34.21% vs 18.71% for GSTT1; 31.58% vs 8.55% for deletion of both genes, in Melanesians and Europeans, respectively) (Table 3). Odds ratios for DTC associated with null as compared with non-null genotypes did not significantly depart from unity in Europeans or in Melanesians, or in both groups combined. No heterogeneity between groups of subjects was detected (Table 3).

**Table 3:** Distribution of *GSTM1* and *GSTT1* genotypes and association with DTC risk by ethnic group.

	Europeans				Melanesians				Europeans and Melanesians				Het
	Cases N = 504	Controls N = 620	OR	95% CI	Cases N = 156	Controls N = 114	OR	95% CI	Cases N = 660	Controls N = 734	OR	95% CI	p-value
	n (%)	n (%)			n (%)	n (%)			n (%)	n (%)			
***GSTM1*** **number of copies**													0.74
0	248 (49.21)	298 (48.06)	0.92	0.60–1.41	124 (79.49)	94 (82.46)	0.80	0.21–3.08	372 (56.36)	392 (53.41)	0.89	0.60–1.34	
1	172 (34.13)	231 (37.26)	0.80	0.52–1.25	25 (16.03)	16 (14.04)	0.82	0.19–3.54	197 (29.85)	247 (33.65)	0.81	0.53–1.24	
≥2	51 (10.12)	53 (8.55)	Ref		6 (3.85)	4 (3.51)	Ref		57 (8.64)	57 (7.77)	Ref	-	
Missing	33 (6.55)	38 (6.13)			1 (0.64)	0 (0)			34 (5.15)	38 (5.18)			
***GSTM1* genotype**													0.49
Null	248 (49.21)	298 (48.06)	1.09	0.85–1.4	124 (79.49)	94 (82.46)	0.94	0.49–1.82	372 (56.36)	392 (53.41)	1.05	0.84–1.33	
Non-null	223 (44.25)	284 (45.81)	Ref		31 (19.87)	20 (17.54)	Ref		254 (38.48)	304 (41.42)	Ref	-	
Missing	33 (6.55)	38 (6.13)			1 (0.64)	0 (0)			34 (5.15)	38 (5.18)			
***GSTT1* number of copies**													0.71
0	90 (17.86)	116 (18.71)	0.81	0.57–1.15	53 (33.97)	39 (34.21)	0.80	0.36–1.75	143 (21.67)	155 (21.12)	0.82	0.60–1.12	
1	196 (38.89)	254 (40.97)	0.80	0.61–1.06	48 (30.77)	44 (38.6)	0.61	0.28–1.33	244 (36.97)	298 (40.6)	0.77	0.59–1.00	
≥2	188 (37.30)	195 (31.45)	Ref		30 (19.23)	16 (14.04)	Ref		218 (33.03)	211 (28.75)	Ref		
Missing	30 (5.95)	55 (8.87)			25 (16.03)	15 (13.16)			55 (8.33)	70 (9.54)			
***GSTT1* genotype**													0.70
Null	90 (17.86)	116 (18.71)	0.92	0.67–1.26	53 (33.97)	39 (34.21)	1.12	0.64–1.97	143 (21.67)	155 (21.12)	0.95	0.73–1.25	
Non-null	384 (76.19)	449 (72.42)	Ref		78 (50)	60 (52.63)	Ref		462 (70)	509 (69.35)	Ref		
Missing	30 (5.95)	55 (8.87)			25 (16.03)	15 (13.16)			55 (8.33)	70 (9.54)			
**Combination of *GSTM1* and *GSTT1***					** **				** **			** **	
***GSTM1/GSTT1* genotype**													0.84
Null/null	41 (8.13)	53 (8.55)	0.97	0.60–1.55	42 (26.92)	36 (31.58)	0.97	0.38–2.50	83 (12.58)	89 (12.13)	0.92	0.62–1.35	
Null/non-null	189 (37.5)	222 (35.81)	1.02	0.77–1.36	62 (39.74)	49 (42.98)	1.06	0.43–2.63	251 (38.03)	271 (36.92)	1.01	0.77–1.32	
Non-null/null	40 (7.94)	52 (8.39)	0.90	0.56–1.44	10 (6.41)	3 (2.63)	3.44	0.69–17.2	50 (7.58)	55 (7.49)	1.03	0.66–1.60	
Non-null/non-null	171 (33.93)	200 (32.26)	Ref		16 (10.26)	11 (9.65)	Ref		187 (28.33)	211 (28.75)	Ref		
Missing	63 (12.5)	93 (15.0)			26 (16.67)	15 (13.16)			89 (13.48)	108 (14.71)			

All OR are adjusted for age, sex, area of residence, and ethnic group where appropriate.

OR: Odds Ratio, CI: confidence interval. Het: test for heterogeneity of odds ratios across ethnic groups

In Table 4, we calculated ORs for BMI, alcohol drinking, and tobacco smoking after stratification of study subjects by *GSTM1* and *GSTT1* genotypes, using a single dataset combining Europeans and Melanesians as we did not detect heterogeneity between the analyses conducted for each group separately (not shown). The association with obesity was greater among individuals with *GSTM1*- and/or *GSTT1*-null genotypes than individuals with non-null genotypes. The OR in obese individuals with null genotype for both genes was 5.75 while it was 1.26 in those with at least one copy of the genes (p-interaction = 0.02). While comparing individuals with null genotype for both genes and BMI≥30 kg/m^2^ to individuals with non-null genotype and BMI<25 kg/m^2^, the OR was 2.69 (p = 0.01) (S1 Table). The inverse association with alcohol intake was particularly noticeable in subjects with *GSTT1*-null genotypes (OR ≥1 glass/week 0.33; p-interaction 0.01) and in those with deletion of both genes (OR≥1 glass/week 0.21; p interaction 0.12). The OR comparing individuals drinking more than one glass per week with non-null genotypes to never drinkers with null genotypes for both genes was 0.36 (p = 0.01) (S1 Table). The association between tobacco smoking and DTC did not differ according to the GSTT1 or GSTM1 genotypes.

**Table 4:** Association between DTC risk and BMI, alcohol consumption, and tobacco smoking stratified by *GSTM1* and *GSTT1* genotypes.

	Null genotype^b^			Non-null genotype			Interaction
	Ca/Co^c^	OR	95% CI	Ca/Co^c^	OR	95% CI	p-value
***GSTM1*** ^**a**^	**N = 366/375**		** **	**N = 249/297**			
**Alcohol drinking**	* *	* *	* *	* *	* *	* *	0.19
Never	130/85	Ref.		76/75	Ref.		
<1 glass/week	171/215	0.57	0.41–0.82	128/151	0.87	0.58–1.31	
≥1 glass/week	65/75	0.70	0.44–1.13	45/71	0.73	0.43–1.24	
**BMI, kg/m^2^**	* *						0.21
≤25	147/185	Ref.		114/160	Ref.		
25–29.9	120/125	1.14	0.80–1.61	77/78	1.38	0.92–2.08	
≥30	99/65	1.78	1.19–2.67	58/59	1.17	0.74–1.86	
**Cigarette smoking**							0.76
Never	213/207	Ref.		133/163	Ref.		
<20 pack-years	108/108	1.08	0.76–1.53	87/90	1.29	0.86–1.95	
≥20 pack-years	45/60	0.80	0.51–1.27	29/44	0.93	0.54–1.63	
***GSTT1***^**a**^	**N = 140/151**			**N = 454/489**			
**Alcohol drinking**							0.01
Never	51/33	Ref.		138/116	Ref.		
<1 glass/week	71/76	0.63	0.35–1.15	216/268	0.74	0.54–1.01	
≥1 glass/week	18/42	0.33	0.15–0.74	100/105	1.01	0.67–1.52	
**BMI, kg/m**^**2**^							0.11
≤25	51/81	Ref.		199/244	Ref.		
25–29.9	48/49	1.96	1.08–3.55	147/149	1.17	0.85–1.59	
≥30	41/21	3.25	1.61–6.55	108/96	1.29	0.91–1.83	
**Cigarette smoking**							0.60
Never	77/75	Ref.		248/271	Ref.		
<20 pack-years	43/49	0.91	0.51–1.62	149/143	1.28	0.94–1.74	
≥20 pack-years	20/27	1.00	0.47–2.13	57/75	0.89	0.59–1.34	
***GSTM1/GSTT1*** ^**a**^	**N = 80/86**			**N = 480/518**			
**Alcohol drinking**							0.12
Never	35/18	Ref.		152/122	Ref.		
<1 glass/week	36/47	0.36	0.16–0.81	238/286	0.71	0.52–0.96	
≥1 glass/week	9/21	0.21	0.07–0.63	90/110	0.83	0.56–1.25	
**BMI, kg/m**^**2**^							0.02
≤25	25/49	Ref.		213/265	Ref.		
25–29.9	25/26	2.14	0.96–4.81	156/152	1.23	0.91–1.66	
≥30	30/11	5.75	2.25–14.7	111/101	1.26	0.89–1.77	
**Cigarette smoking**							0.52
Never	49/45	Ref.		263/290	Ref.		
<20 pack-years	21/26	0.90	0.40–2.00	161/152	1.31	0.96–1.76	
≥20 pack-years	10/15	0.80	0.27–2.41	56/76	0.89	0.60–1.34	

^a^ The multivariate regression model includes age, sex, area of residence, ethnic group, BMI, alcohol consumption, and cigarette smoking.

^b^ Null genotype refers to individuals with null genotypes for both genes, while non-null genotype refers to subjects with at least one copy of *GSTM1* or *GSTT1*

^c^The number of subjects do not add up to the total because of missing values for some variables

Ca/Co: Cases/Controls, OR: Odds ratio, 95% CI: 95% confident Interval, *p-int*: p-value of interaction

The analyses for hormonal and reproductive factors in women stratified by genotypes are shown in Table 5. No significant interaction with GSTM1 was detected for age at menarche, parity, oral contraceptive, or menopausal status. Late age at menarche was positively associated with DTC in individuals with GSTT1 non-null genotype for but not in those with GSTT1-null genotype (p interaction 0.03).

**Table 5:** Association between DTC risk and hormonal and reproductive factors stratified by *GSTM1* and *GSTT1* genotypes in women.

	Null genotype^b^			Non-null genotype^b^			Interaction^c^
	Ca/Co	OR	95% CI	Ca/Co	OR	95% CI	p
***GSTM1***^**a**^	**N = 289/254**		*** ***	**N = 196/207**			
**Age at menarche**		** **	** **		** **	** **	0.86
≤13 years	206/199	Ref.		152/170	Ref.		
>13 years	83/55	1.41	0.91–2.19	44/37	1.31	0.77–2.24	
**Oral contraception use**			** **			** **	0.14
Never	123/100	Ref.		65/49	Ref.		
Ever	166/154	1.05	0.65–1.68	131/158	0.58	0.33–1.02	
**Parity**			** **			** **	0.35
Nulliparous	28/26	Ref		15/26	Ref		
Parous	261/228	1.15	0.61–2.16	181/181	1.98	0.94–4.16	
**Menopausal status**			** **			** **	0.52
Pre-menopause	147/140	Ref.		88/113	Ref.		
Post-menopause	142/114	0.93	0.52–1.68	108/94	2.67	1.24–5.78	
***GSTT1***^**a**^	**N = 106/104**			**N = 352/326**			
**Age at menarche**			** **			** **	0.03
≤13 years	78/71	Ref.		263/275	Ref.		
>13 years	28/33	0.82	0.39–1.69	89/51	1.80	1.19–2.72	
**Oral contraception use**			** **			** **	0.41
Never	50/49	Ref.		122/89	Ref.		
Ever	56/55	0.97	0.42–2.26	230/237	0.78	0.51–1.19	
**Parity**			** **			** **	0.23
Nulliparous	10/16	Ref.		30/29	Ref.		
Parous	96/88	2.02	0.75–5.49	322/297	1.15	0.64–2.07	
**Menopausal status**			** **			** **	0.42
Pre-menopause	56/55	Ref.		168/181	Ref.		
Post-menopause	50/49	0.91	0.34–2.47	184/145	1.49	0.86–2.60	
***GSTM1/GSTT1***^**a**^	**N = 61/65**		** **	**N = 387/357**			
**Age at menarche**			** **			** **	0.09
≤13 years	45/44	Ref.		289/294	Ref.		
>13 years	16/21	0.77	0.29–2.06	98/63	1.54	1.04–2.27	
**Oral contraception use**			** **			** **	0.70
Never	34/35	Ref.		133/101	Ref.		
Ever	27/30	0.95	0.32–2.80	254/265	0.82	0.55–1.22	
**Parity**			** **			** **	0.45
Nulliparous	6/10	Ref.		31/35	Ref.		
Parous	55/55	2.01	0.50–8.08	356/322	1.30	0.76–2.25	
**Menopausal status**			** **			** **	0.48
Pre-menopause	34/35	Ref.		185/196	Ref.		
Post-menopause	27/30	1.02	0.27–3.85	202/161	1.46	0.86–2.48	

^a^ The multivariate regression model included age, sex, area of residence, ethnic group, BMI, alcohol consumption, cigarette smoking, age at menarche, number of full-term pregnancies, and oral contraception.

^b^ Null genotype refers to individuals with null genotypes for both genes, whereas non-null genotype refers to subjects with at least one copy of *GSTM1* or *GSTT1*.

^c^ Test for the interaction between the corresponding variables and the genotypes

Ca/Co: cases/controls, OR: odds ratio, CI: confidence interval

## Discussion

This study was conducted in two populations with different genetic background and distinct patterns of exposure to environmental factors. The associations we reported between DTC and lifestyle, hormonal and reproductive factors are consistent with those published previously in the total samples of NC and CATHY studies [7, 18, 19]. We showed that BMI and alcohol consumption were more strongly associated with DTC in individuals with *GSTM1* and *GSTT1*-null genotypes than in those with non-null genotypes. A significant interaction between *GSTT1*-null and age at menarche was also observed. By considering genes and environmental or lifestyle factors, no heterogeneity between groups was observed.

### CNV distribution in Europeans and Melanesians

The distribution of *GSTM1* and *GSTT1* CNVs among Europeans in our study was similar to previous reports [20,21]. To the best of our knowledge, this is the first report of CNV distribution in Melanesians. We found that the frequency of null genotypes was notably higher in Melanesians than in Europeans, which is consistent with previous data concerning other Pacific islanders [22].

### Interaction with BMI

Being overweight has consistently been associated with DTC risk [23,24]. In the present study, obesity was more strongly associated with DTC in *GST-null* than non-null individuals, particularly when both genes were deleted. This finding was consistent in both ethnic groups. To the best of our knowledge, the only study to investigate the joint effect of BMI and GST genes in DTC was conducted in Korea and found no indication of an interaction [25]. However, the prevalence of obesity in this population is very low and the study may have been underpowered. Studies in populations with a high prevalence of obesity would be helpful for confirming our finding.

Obesity may increase the risk of DTC through complex mechanisms [26]. The chronic systemic inflammation induced by obesity may favor cancer development through the formation of reactive oxygen species (ROS) [27]. Because the enzymes encoded by *GSTM1* and *GSTT1* contribute to reducing oxidative stress by conjugating and eliminating ROS products [28], obese subjects with deletion of these genes could be at particularly high risk of DTC.

### Interaction with alcohol drinking and tobacco smoking

The observed inverse association of alcohol drinking with DTC is in line with several studies [10,11,29–31] and a recent meta-analysis [32]. This inverse association was greater in *GSTM1*/*GSTT1*-null subjects than non-null subjects, particularly in those with a deletion of both genes. An interaction between alcohol intake and *GSTM1* or *GSTT1* was reported previously in cancer of the breast, lung, and stomach [33–35]. Our study is the first report of such an interaction in thyroid cancer.

The mechanisms underlying the potentially protective effect of alcohol drinking in thyroid carcinogenesis are not known. Some studies have suggested that free radicals generated by alcohol metabolism may have either a direct toxic effect on the thyroid tissues or disturb the hypothalamus–pituitary–thyroid axis [30,36,37]. However, how this can lead to a decreased risk of DTC remains unclear [38], and it does not explain the synergistic effect of alcohol and GST-null genotypes.

Unlike alcohol, *GSTM1* and *GSTT1* were not found to modify the association between tobacco smoking and DTC. The interaction between GST genes and smoking was also investigated in a previous study, but no evidence of an interaction was reported [22].

### Interaction with hormonal and reproductive factors

Given the higher incidence of DTC in women than in men, female sex hormones have been suspected to play a major role in thyroid carcinogenesis [39]. We found that late age at menarche was associated with increased DTC incidence. This association was also reported by several studies [7,40,41], but a recent meta-analysis found no conclusive evidence in cohort studies [42]. We also found that ever users of oral contraceptives were at decreased risk, a finding supported by recent studies [43,44]. Conversely, parity was not associated with DTC in our data. There was also some indication that menopause increases DTC risk. These findings were described in detail previously in the CATHY [18] and NC [7,19] studies.

Our study is the first to investigate the interaction between GST genotype and reproductive factors in thyroid cancer. No interaction was observed between *GSTM1* and age at menarche, use of oral contraceptives, parity, or menopausal status, whereas significant interaction was observed between *GSTT1* and age at menarche.

The association of DTC with age at menarche has been inconsistent in epidemiological studies, and the potential underlying mechanisms involved are unclear. It has been shown that expression of *GSTM1* differs during menstruation [39] and polymorphisms in the GSTs genes were associated to various endometrial pathologies such as for instance endometriosis [45]. However, how *GSTT1* may modulate the association between late age at menarche and DTC risk remains to be elucidated.

### Strengths and limitations

This study used data collected from two case-control studies in Europeans and Melanesians with a population-based design and exhaustive identification of thyroid cancer cases. Our study was based on a relatively high number of subjects. With the exception of one study in Korea that included 1372 cases and 1669 controls [25], previous studies of DTC risk in relation to *GSTM1* or *GSTT1* genotype have had limited sample sizes of less than 300 cases. Unlike previous studies contrasting DTC risk in null vs. non-null genotypes, CNVs of *GSTM1* and *GSTT1* were also examined in our study to investigate a potential dose-effect relationship between enzymatic activity and cancer risk. However, such a dose-effect association was not observed in our data. We also examined gene-environment interactions between *GSTM1* and *GSTT1* genotypes and suspected risk factors of DTC. The analyses of risk factors in these two ethnic groups with diverse prevalence of exposure to risk factors and frequency of GST-null genotypes could help explain the difference in DTC incidence between the study populations. In particular, the highest prevalence of both GST-null genotypes and obesity in Melanesians compared to Europeans could partly explain the higher incidence of DTC in Melanesians.

One limitation of our study is the limited statistical power in some strata of the stratified analyses. Potential recall bias inherent to case-control studies may have occurred when assessing exposure to several risk factors. But differential misclassification was unlikely as cases and controls were interviewed in the same conditions by trained interviewers. Also, we cannot exclude that some of the associations found are due to the number of tests used. Replication in independent studies are necessary to confirm our results.

In conclusion, our results suggest that *GSTM1* and *GSTT1* may modify the associations between DTC risk and obesity, alcohol consumption, and possibly hormonal factors. Disparities in the frequency of *GSTM1* and *GSTT1* deletion and in the prevalence of obesity between populations worldwide, may partly explain differences in thyroid cancer incidence.

## Supporting information

S1 Table Association between DTC risk and combination of GST genotypes and BMI, alcohol consumption and tobacco smoking variables. (XLSX)
